# Positive Effects of Lycopene on Growth Performance, Hepatic Antioxidant Capacity, Intestinal Morphology, and Cecal Microflora of Yellow-Feather Broilers

**DOI:** 10.3390/ani15142108

**Published:** 2025-07-16

**Authors:** Guangtian Cao, Xiyue Liu, Huixian Wang, Jia Kang, Feiyang Wang, Molin Li, Wenqi Sun, Xiaosi Lv

**Affiliations:** 1 College of Quality and Standardisation, China Jiliang Universtiy, Hangzhou 310058, China; 15a1903025@cjlu.edu.cn (G.C.); cleanp1@163.com (F.W.); bo3199zhijie@163.com (M.L.); 19563399863@139.com (W.S.); 2 Zhejiang Jialemei Food Co., Ltd., Huzhou 313307, China; 3 Department of Life Sciences, Imperial College London, London SW7 2AZ, UK; lxyhnyxl6@gmail.com; 4 Key Laboratory of Applied Technology on Green-Eco-Healthy Animal Husbandry of Zhejiang Province, Zhejiang Provincial Engineering Laboratory for Animal Health and Internet Technology, College of Animal Science and Technology, Zhejiang A & F University, Hangzhou 311300, China; 18864833071@163.com (H.W.); hnkjhh@163.com (J.K.); 5 Zhejiang Institute of Quality Sciences, Hangzhou 310018, China

**Keywords:** lycopene, yellow-feather broilers, antioxidant capacity, intestinal morphology, cecal microflora

## Abstract

Lycopene (Lyc) possesses strong antioxidant activity through its ability to scavenge singlet oxygen and modulate immune responses in livestock and poultry. As few studies have examined the effects of Lyc on hepatic antioxidant capacity in broilers, we evaluated the effects of dietary Lyc supplementation on growth performance, antioxidant capacity, intestinal morphology, and microflora composition in yellow-feather broilers. We randomly assigned 480 one-day-old yellow-feather broilers to four dietary treatments: a basal diet with no additive (Con), and a basal diet supplemented with 150 mg/kg (Lyc150), 250 mg/kg (Lyc250), or 500 mg/kg (Lyc500) of lycopene. Compared to Con, Lyc supplementations significantly increased average daily gain by more than 14% from days 29 to 56. Additionally, Lyc250 significantly increased by over 6.4% in average daily feed intake from days 1 to 28, as well as by over 50% in hepatic levels of total antioxidant capacity and 65% in catalase on day 56. Lyc250 significantly increased villus height and decreased the villus-to-crypt ratio. Moreover, both Lyc250 and Lyc500 significantly downregulated hepatic Bax mRNA expression. Further, Lyc150 and Lyc250 significantly increased the relative abundance of *Fermentum_g_Lactobacilus*, *Enterococcus_cecorum_g_Enterococcus*, and *Ruminococcus sp_g_Ruminococcus*, but decreased *Acetobacter_lovaniensis_g_Acetobacter* and *Lactobacillus_amylolyicus_g_Lactobacilus*. Overall, our study found that Lyc supplementation significantly improved growth performance, enhanced hepatic antioxidant capacity, and modulated cecal microflora through increasing beneficial species in yellow-feather broilers.

## Simple Summary

Recently, plant extracts have been confirmed to effectively inhibit diseases in poultry production, especially with the prohibition of antibiotics. Plant-derived compounds have also been shown to improve the growth performance of broilers owing to their biological functions. We suspected that the lycopene, treated as one of the most potent antioxidants, may beneficially contribute to the broilers, and we arranged the present trial based on the differential addition dosages. Data confirmed the growth promotion of lycopene through improving intestinal morphology, enhancing hepatic antioxidant capacity, and increasing beneficial cecal species. Hence, we suggested that lycopene is one kind of profitable plant extract, which may beneficially improve the growth performance of yellow-feather broilers.

## 1. Introduction

With the prohibition of antibiotics, plant extracts have been recognized for their protective effects in animals by effectively inhibiting diseases and alleviating economic stress [1]. Plant-derived compounds, such as carotenoids, isoflavones, and polyphenols, have been shown to improve the growth performance of broilers, owing to their diverse molecular structures and biological functions [2]. Among them, plant-derived carotenoids, which are pure hydrocarbons that serve as the main dietary source of provitamin A, are of particular interest [3]. As precursors of vitamin A, carotenoids play a role in modulating the innate immune system of birds [4]. Dietary carotenoids can also autocatalytically produce complex oxygen copolymers, which stimulate innate immune responses and help counteract inflammation [5].

Lycopene (Lyc), a red pigment found in tomato, is a polyunsaturated aliphatic hydrocarbon [6,7]. Recognized as one of the most potent antioxidants, lycopene is classified as a Class A nutrient due to its ability to neutralize singlet oxygen [8,9]. Studies have shown that β-carotene improves the growth performance of Ross 308 broilers in terms of parameters such as final body weights, feed intake, average daily gain, and feed conversion [10]. Lyc administration has also been shown to enhance the antioxidant capacity and immune function of broilers [11,12]. Additionally, Lyc has been reported to activate the antioxidant defense pathway of Nrf2 [13]. Wu [2] revealed that Lyc beneficially increased jejunum villous height and glutathione peroxidase enzyme concentration and modulated cecal microflora. Zhao [14] found that Lyc alleviates dextran sulfate sodium salt-induced colitis by modulating the microbe–gut–brain axis balance. Moreover, dietary lycopene has been shown to alleviate intestinal damage and impairment in immune and barrier functions caused by aflatoxin B1 and reduce oxidative stress in broiler chickens [15]. However, few studies have focused on the effects of Lyc on hepatic antioxidant capacity in broilers.

Hence, this study aimed to investigate the effects of Lyc on growth performance, intestinal morphology, hepatic antioxidant status, and cecal microflora in yellow-feather broilers.

## 2. Materials and Methods

### 2.1. Animals, Feeding, and Experimental Design

Overall, 480 one-day-old yellow-feather broilers were randomly assigned to 4 treatment groups (6 replicates with 20 birds per cage): the Con group, receiving a basal diet without any additive (Con), and three experimental groups, receiving basal diets supplemented with 150, 250, and 500 mg/kg Lyc. Throughout the 56-day trial, all birds had ad libitum access to water and feed, and the birds received two-stage feeding as listed in Table 1. And the differential dosage of Lyc was evenly mixed into the feed of the experimental groups before the trials. Standard management practices, including vaccination and sanitary protocols, were maintained according to typical poultry house conditions. For the first week, the house temperature of all birds was maintained at 34 °C and was then gradually decreased to 27 °C at a rate of 2 °C/week. On day 56, one bird per cage was selected for further sampling and analysis, with a total of twenty-four birds. The blood samples were collected from the wing vein, and the birds were euthanized after that. Then, the serum was separated and stored at −20 °C for immune response analysis. After that, the left liver samples were collected and stored at −20 °C for antioxidant assays. Sections of the ileum and liver were preserved in 10% formaldehyde for hematoxylin–eosin (H&E) staining and scanning electron microscopy (SEM) analysis. Cecal contents were collected in sterile freezing tubes and stored at −80 °C for 16S rRNA high-throughput sequencing. Lyc consisted of 20% trans-lycopene and 80% maltodextrin, which were purchased from Chenguang Biotech Group Co., Ltd. (Handan, China). The composition and nutritional content of the basal experimental diet are listed in Table 1, which follows the National Research Council guidelines [16].

### 2.2. Growth Performance

Birds were weighed by platform (Suhnsh, Shanghai, China) on days 1, 21, and 56 to calculate the average daily gain (ADG). Feed intake per cage was recorded weekly to calculate the average daily feed intake (ADFI) and the feed/gain (F:G) ratio.

### 2.3. Serum Immune Responses

Serum concentrations of IgA, IgM, IgY, IL-1β, IL-8, IL-10, and TNF-α were measured by an infinite^®^ F50 microplate reader (Tecan, Männedorf, Switzerland) using specific commercial ELISA kits (Aoqing Biotechnology, Nanjing, China), strictly following the manufacturer’s protocols.

### 2.4. Hepatic Antioxidant Capacity

Hepatic antioxidative parameters, such as total antioxidant capacity (T-AOC), catalase (CAT), superoxide dismutase (SOD), malondialdehyde (MDA), and glutathione peroxidase (GSH-Px), were determined by the multiskan^TM^ GO microplate spectrophotometer (Thermo Fisher Scientific, Vantaa, Finland) using commercial assay kits (Aoqing Biotechnology, Nanjing, China), which were used in strict accordance with the manufacturer’s instructions.

### 2.5. Ileal Morphology

After fixation, dehydration, and paraffin embedding, a 0.5 cm section of the ileum was fixed in 10% formaldehyde (Shanghai Aladdin Biochemical Technology Co., Ltd., Shanghai, China) and stained with H&E. Histological images of the ileal structure were captured using a fluorescence microscope (DS-FI2 camera, EclipseCi, Nikon, Tokyo, Japan). Ten villi per sample were selected to calculate the villus height, crypt depth, and villus height/crypt depth ratio.

Transmission electron microscopy (TEM) was used to assess ileal morphology, following stabilization, post-stabilization, and dehydration procedures described in our previous study [17]. The ileal tissues were sectioned using Leica A-1170 (Leica, Wetzlar, Germany), stained with 2% uranium acetate and lead citrate, and examined using SEM (JEOL2100 HR, Hitachi, Tokyo, Japan).

### 2.6. Hepatic Morphology and Apoptosis-Related Gene Expression

Approximately 1.0 g of liver tissue was fixed in 10% formaldehyde, stained with H&E, and examined using a fluorescence microscope (DS-FI2 camera, EclipseCi, Nikon, Tokyo, Japan).

For gene expression analysis, the hepatic sample was ground into a powder in a mortar filled with liquid nitrogen, violently shaken at 25 °C with chloroform, and then centrifuged at 12,000 rpm at 25 °C. The supernate was then transferred to a new centrifuge tube, and isopropanol was added. After centrifugation (4 °C, 12,000 rpm, 10 min), pre-cooled ethanol was added to the sediment to dry in a clean bench. The sample was dissolved using diethypyrocarbonate and then reverse-transcribed using a specific kit (ABClonal, Wuhan, China). The gel electrophoresis system (CPS300, Tanon, Fort Lauderdale, FL, USA), quantitative real-time PCR (CFX96, Bio-Rad, Hercules, CA, USA), and DNA gel imaging system (2500, Tanon, USA) were used for detecting gene expression. And, the 2^−ΔΔ^Ct method was used for calculating the relative abundance of the genes of interest, as listed in Table 2, where GADPH was treated as the internal standard. The primers for apoptosis-related genes are shown in Table 2.

### 2.7. Cecal Short-Chain Fatty Acids

High-performance gas chromatography was used for the determination of short-chain fatty acids (SCFAs) according to our previous study [18]. Approximately 0.5 g of cecal digesta per sample was mixed with ddH_2_O (1:1, *v*/*v*) and centrifuged at 12,000 rpm at 4 °C for 10 min, and the supernatant was then blended with 25% metaphosphoric acid in an ice bath for 30 min. After a second centrifugation, the supernatant was screened with a 0.22 μm filter and analyzed using an Agilent Technologies 7890B GC System (column dimensions: 30 m × 0.25 mm × 0.25 μm; Agilent Technologies, Santa Clara, CA, USA).

### 2.8. Cecal Microflora Structure

Genomic DNA from 24 cecal samples was extracted using the MagPure Soil DNA LQ kit following the manufacturer’s instructions (Magen, Guangzhou, China). The V3-V4 variable regions of the 16S rRNA genes were amplified with universal primers 343 F (5′-TACGGRAGGCAGCAG-3′) and 798 R (5′-AGGGTATCTAATCCT-3′). After the library construction, sequencing and bioinformatic analyses were conducted by OE Biotech Co., Ltd. (Shanghai, China). Briefly, the raw sequencing data in a FASTQ format were preprocessed using the Cutadapt software (version 5.0) for adapter trimming and quality filtering. Chimera reads were removed using DADA2 (version 1.4) with the default parameters of QIIME2 (2020.11). All representative reads were annotated and blasted against the Silva database version 138. Operational taxonomic units (OTUs) were clustered at 97% similarity using Vsearch (version 2.4.2) software. Microbial diversity was assessed via α-diversity indices (Shannon and Goods_coverage) and β-diversity measures [(principal component analysis (PCA) and principal coordinate analysis (PCoA)]. Differential microbial taxa at the genus level were identified using the Kruskal–Wallis test and linear discriminant analysis effect size (LEfSe; LDA value was set at 2.0).

### 2.9. Statistical Analysis

The SPSS 26 software (SPSS Inc., Chicago, IL, USA) was used for statistical data analysis. A one-way analysis of variance (ANOVA) was used to analyze the growth performance, hepatic antioxidant capacity, serum immune responses, intestinal morphology, and SCFAs. Significant differences were considered at *p* < 0.05, and highly significant differences were considered at *p* < 0.01. Graphs were generated using Graphpad Prism 8.0 (GraphPad Prism Inc., San Diego, CA, USA).

## 3. Results

### 3.1. Growth Performance

The effects of Lyc supplementation on the growth performance of yellow-feather broilers are shown in Table 2. From days 29 to 56, both Lyc treatments significantly increased (*p* < 0.001, *p* < 0.001, and *p* = 0.011) the ADG of birds compared to Con. Moreover, the Lyc250 treatment significantly improved (*p* = 0.008) the ADFI of birds from days 1 to 28 compared to Con (Table 3). Although the Lyc treatment decreased the F:G of birds compared with Con through the whole trial period, the differences were not statistically significant.

### 3.2. Hepatic Antioxidant Capacity

**Figure 1:**
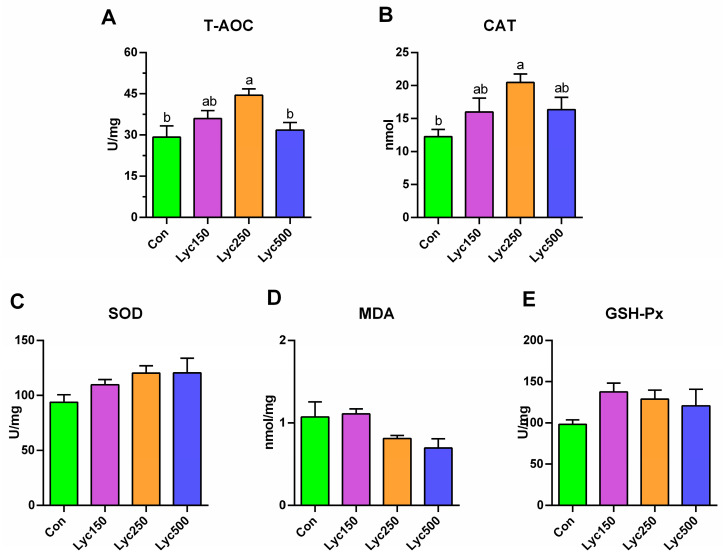
Effects of lycopene on the hepatic antioxidant capacity of yellow-feather broilers: (**A**) represents T-AOC, (**B**) represents CAT, (**C**) represents SOD, (**D**) represents MDA, and (**E**) represents GSH-Px. Con represents birds fed a basal diet, Lyc150 represents birds fed a basal diet with 150 mg of lycopene/kg added, Lyc250 represents birds fed a basal diet with 250 mg of lycopene/kg added, and Lyc500 represents birds fed a basal diet with 500 mg of lycopene/kg added. Different lowercase letters represent significantly different means, *p* < 0.05. *n* = 6 per group.

Lyc250 supplementation significantly increased (*p* < 0.001) hepatic T-AOC and CAT levels compared to Con; however, the other Lyc supplementation showed a numerical increase in these two parameters (Figure 1A,B). No significant differences were observed in the concentration of SOD, MDA, and GSH-Px among all treatments; however, SOD and GSH-Px levels were numerically elevated in the Lyc treatment group (Figure 1C–E).

### 3.3. Immune Parameters

**Figure 2:**
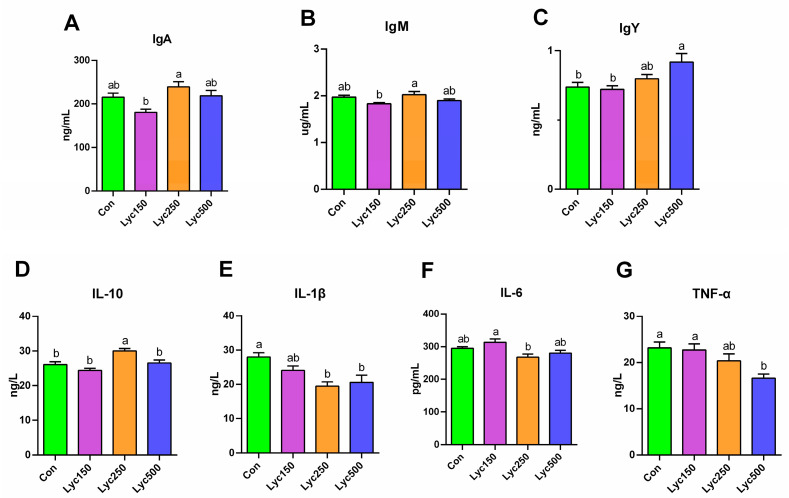
Effects of lycopene on the serum immune responses of yellow-feather broilers: (**A**) represents IgA, (**B**) represents IgM, (**C**) represents IgY, (**D**) represents IL-10, (**E**) represents IL-1β, (**F**) represents IL-6, and (**G**) represents TNF-α. Con represents birds fed a basal diet, Lyc150 represents birds fed a basal diet with 150 mg of lycopene/kg added, Lyc250 represents birds fed a basal diet with 250 mg of lycopene/kg added, and Lyc500 represents birds fed a basal diet with 500 mg of lycopene/kg added. Different lowercase letters represent significantly different means, *p* < 0.05. *n* = 6 per group.

The concentrations of serum IgA, IgM, and IgY were numerically increased by Lyc250 supplementation compared to Con, though the differences were not statistically significant (Figure 2A–C). However, Lyc500 treatment significantly increased (*p* < 0.05) serum IgY levels, and Lyc250 treatment significantly increased the level of IL-10 and decreased IL-1β levels (Figure 2D,E) compared to Con. Both Lyc supplementations decreased the IL-1β and TNF-α levels compared to Con, but these differences were not significant (Figure 2E–G). Furthermore, the Lyc250 and Lyc500 treatments numerically decreased the concentration of IL-6, whereas Lyc150 showed a numerical increase in IL-6 compared to Con (Figure 2F).

### 3.4. Intestinal Morphology

**Figure 3:**
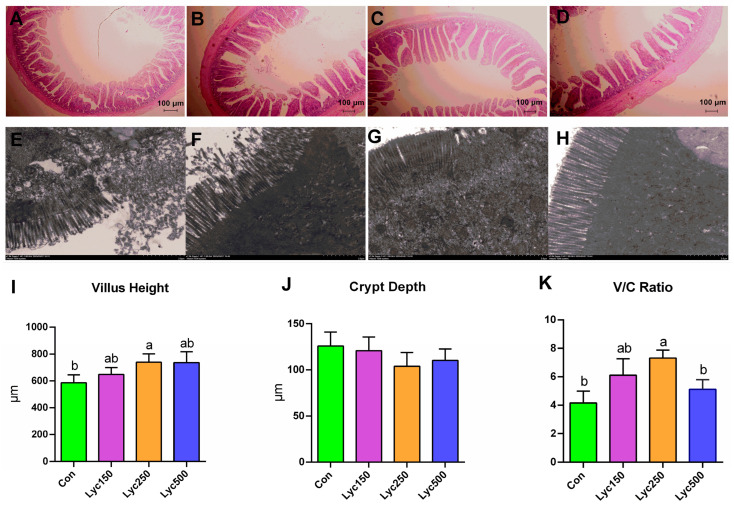
Effects of lycopene on the ileal morphology of yellow-feather broilers: (**A**–**D**) ileal H&E stains of Con, Lyc150, Lyc250, and Lyc500; (**E**–**H**) ileum TEM figures of Con, Lyc150, Lyc250, and Lyc500; (**I**–**K**) villus height, crypt depth, and villus height/crypt depth ratio. Con represents birds fed a basal diet, Lyc150 represents birds fed a basal diet with 150 mg of lycopene/kg added, Lyc250 represents birds fed a basal diet with 250 mg of lycopene/kg added, and Lyc500 represents birds fed a basal diet with 500 mg of lycopene/kg added. Different lowercase letters represent significantly different means, *p* < 0.05. *n* = 6 per group.

H&E staining revealed that intestinal villi integrity was improved in Lyc250 and Lyc500 treatment groups (Figure 3A–D); the TEM images indicated more structurally intact mitochondria in the ileal tissues of Lyc-supplemented birds (Figure 3E–H); and Lyc250 supplementation significantly increased (*p* < 0.05) the ileal micro-villus height and decreased the villus-to-crypt (V/C) ratio (Figure 3I,K), all in comparison to Con. Although both Lyc250 and Lyc500 treatments decreased the crypt depth compared to Con, there was no significant difference (Figure 3J).

### 3.5. Hepatic Morphology and Oxidative Damage to mRNA Expression

**Figure 4:**
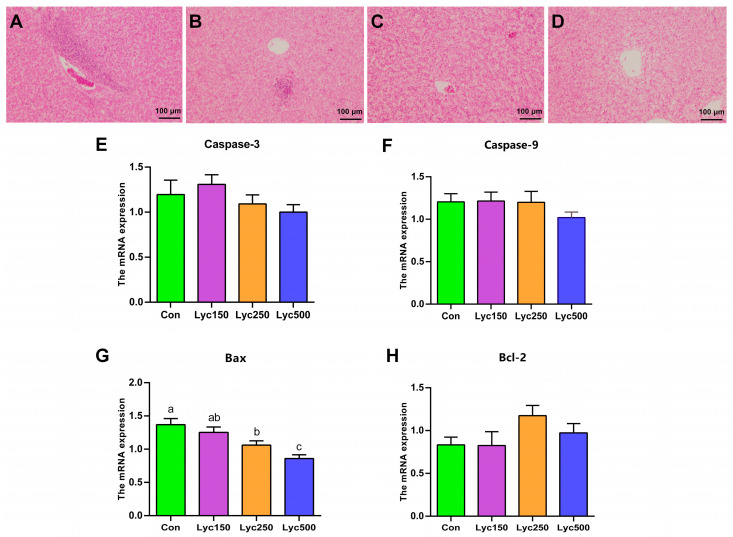
Effects of lycopene on the hepatic morphology and oxidative damage to the mRNA expression of yellow-feather broilers: (**A**–**D**) hepatic H&E stains of Con, Lyc150, Lyc250, and Lyc500; (**E**–**H**) the mRNA expression of caspase-3, caspase-9, Bax, and Bcl-2. Con represents birds fed a basal diet, Lyc150 represents birds fed a basal diet with 150 mg of lycopene/kg, Lyc250 represents birds fed a basal diet with 250 mg of lycopene/kg, and Lyc500 represents birds fed a basal diet with 500 mg of lycopene/kg added. Different lowercase letters represent significantly different means, *p* < 0.05. *n* = 6 per group.

H&E staining showed a reduced infiltration of inflammatory hepatic cells in the livers of Lyc-treated birds compared to that in Con birds (Figure 4A–D). No significant differences were observed in the mRNA expression levels of hepatic caspase-3, caspase-9, and Bcl-2 among the groups (Figure 4E,F,H). However, Lyc250 and Lyc500 treatments significantly decreased Bax mRNA expression compared to Con (Figure 4G). Although Lyc150 and Lyc500 supplementation showed a numerical increase in the mRNA expression of caspase-9 compared to Con, there was no significant difference.

### 3.6. Cecal SCFAs

**Figure 5:**
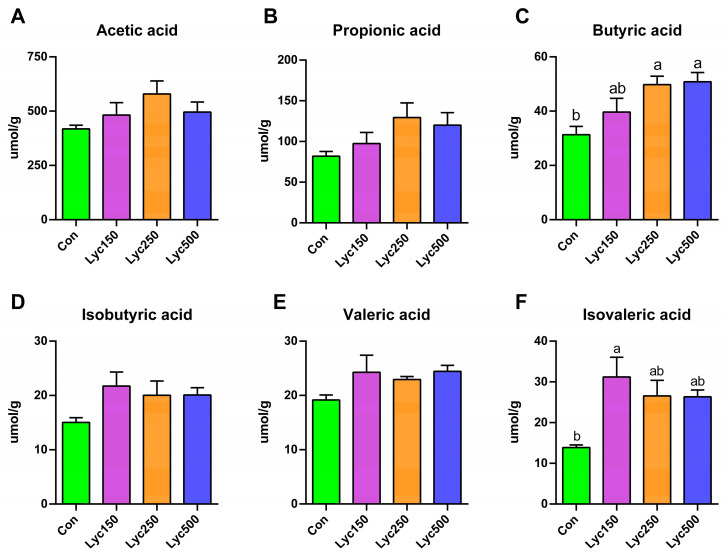
Effects of lycopene on cecal SCFAs in yellow-feather broilers: (**A**) acetic acid, (**B**) propionic acid, (**C**) butyric acid, (**D**) isobutyric acid, (**E**) valeric acid, and (**F**) isovaleric acid. Con represents birds fed a basal diet, Lyc150 represents birds fed a basal diet with 150 mg of lycopene/kg added, Lyc250 represents birds fed a basal diet with 250 mg of lycopene/kg added, and Lyc500 represents birds fed a basal diet with 500 mg of lycopene/kg added. Different lowercase letters represent significantly different means, *p* < 0.05. *n* = 6 per group.

Lyc250 and Lyc500 treatments significantly increased the concentration of cecal butyric acid (Figure 5C), and isovaleric acid levels were significantly increased by Lyc150 treatment (Figure 5F) compared to Con. Although the levels of acetic acid, propionic acid, isobutyric acid, and valeric acid were more elevated in Lyc treatment groups than in Con, there was no significant difference (Figure 5A,B,D,E).

### 3.7. Cecal Microflora

**Figure 6:**
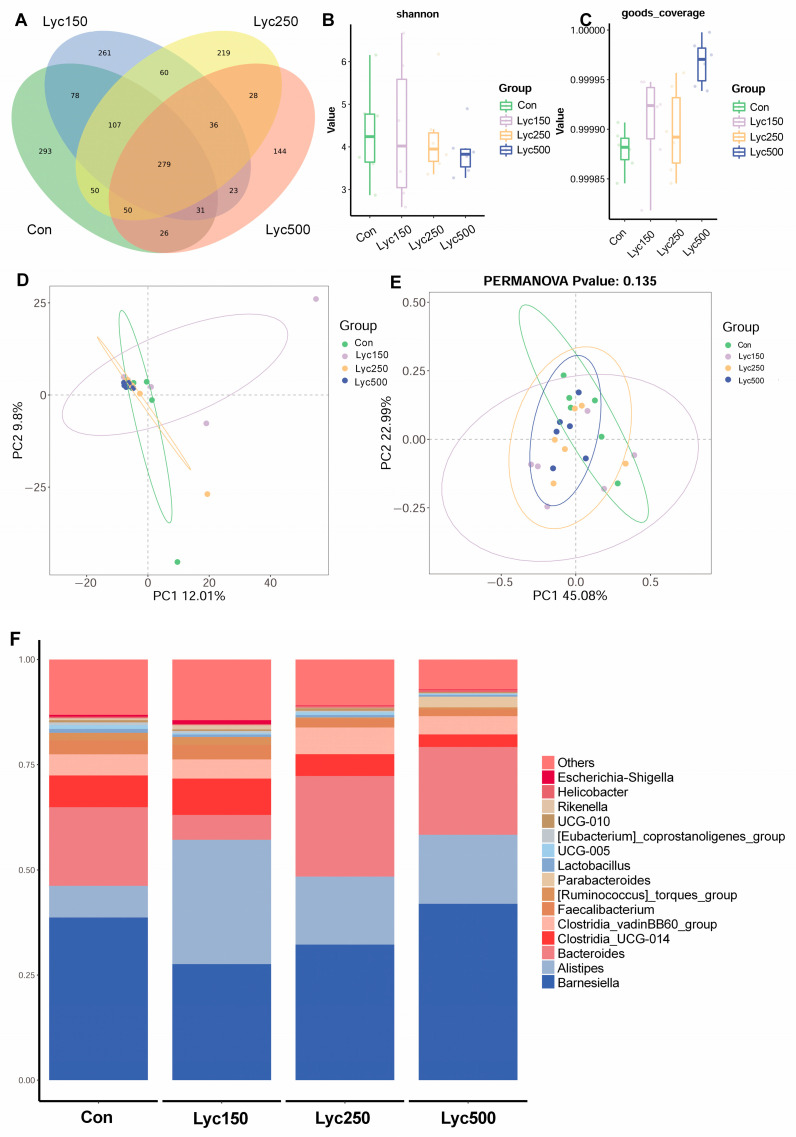
Effects of lycopene on the high-throughput sequencing of cecal content in yellow-feather broilers: (**A**) Venn diagram; (**B**) Shannon index; (**C**) goods_coverage index; (**D**) PCA plot based on genus level; (**E**) PCoA plot based on genus level; (**F**) the relative abundance of the top 15 genera. Con represents birds fed a basal diet, Lyc150 represents birds fed a basal diet with 150 mg of lycopene/kg added, Lyc250 represents birds fed a basal diet with 250 mg of lycopene/kg added, and Lyc500 represents birds fed a basal diet with 500 mg of lycopene/kg added. *n* = 6 per group.

The Venn diagram figure shows that the Con, Lyc500, Lyc250, and Lyc500 birds had 293, 261, 219, and 144 unique sequences, respectively (Figure 6A). No significant differences were observed in goods_coverage or the Shannon index (alpha-diversity) among treatment groups; however, Lyc supplementation treatments increased the goods_coverage compared with Con (Figure 6B,C). Moreover, there was no significant difference between the Simpson index and the Chao1 index, meaning that there was no obvious difference in the richness and evenness of cecal microflora (Supplemental Figure S1A,B). The PCA and PCoA plots indicated distinct clustering of the samples from Lyc-treated groups, suggesting a microbial community shift (Figure 6D,E). *Barnesiella*, *Alistipes*, *Bacteroides*, *Clostridia_UCG−014*, *Clostridia_vadinBB60_group*, *Faecalibacterium*, *[Ruminococcus]_torques_group*, *Parabacteroides*, *Lactobacillus*, and *UCG−005* were the top 10 genera of birds’ microflora (Figure 6F).

**Figure 7:**
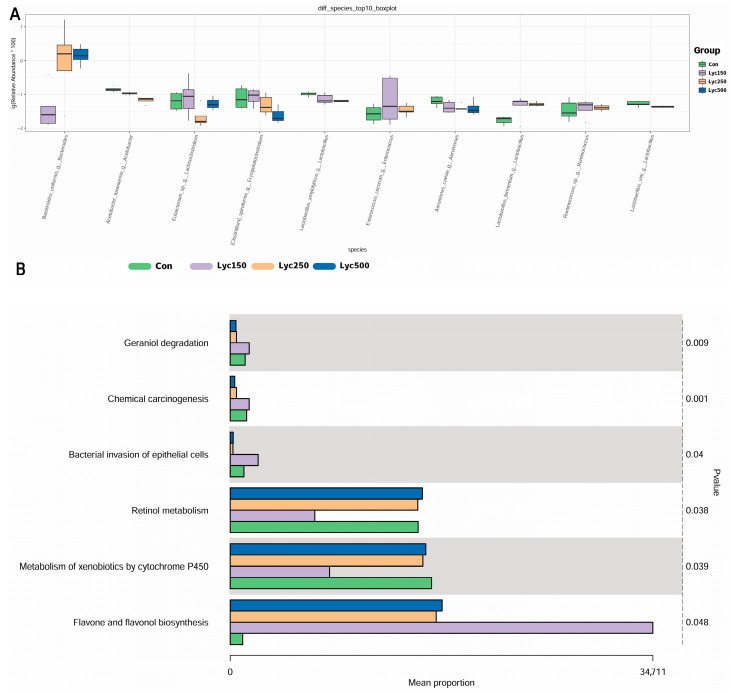
Effects of lycopene on multi-analysis and KEGG metabolic pathways of yellow-feather broilers’ cecal microflora: (**A**) multi-analysis based on the species level; (**B**) KEGG metabolic pathway mean proportion on level 3. Con represents birds fed a basal diet, Lyc150 represents birds fed a basal diet with 150 mg of lycopene/kg added, Lyc250 represents birds fed a basal diet with 250 mg of lycopene/kg added, and Lyc500 represents birds fed a basal diet with 500 mg of lycopene/kg added. *n* = 6 per group.

The top 10 significantly changed microbial species across all bird treatments are shown in Figure 7A. Compared with Con, Lyc supplementation significantly increased the relative abundance of *Bacteroides_uniformis_g_Bacteroides* and decreased that of *Acetobacter_lovaniensis_g_Acetobacter*, *Aeromonas_caviae_g_Aeromonas*, and *Lactobacillus_vini_g_Lactobacillus*. Both Lyc150 and Lyc250 significantly increased *Lactobacilus fermentum_g_Lactobacilus*, *Enterococcus_cecorum_g_Enterococcus*, and *Ruminococcus sp_g_Ruminococcus* and decreased *Acetobacter_lovaniensis_g_Acetobacter* and *Lactobacillus_amylolyicus_g_Lactobacilus*. The Lyc treatment significantly increased the pathway of Flavone and flaconol biosynthesis, and it significantly downregulated the expression of geraniol degradation, chemical carcinogenesis, and the bacterial invasion of epithelial cells compared with Con, except for the lowest dosage, Lyc150 (Figure 7B).

## 4. Discussion

Lycopene, a major extract from tomatoes, has recently become a focal point of nutrition and physiological research in poultry. Polyphenols have been shown to enhance the growth performance of broilers, in terms of parameters such as body weight, body weight gain, and feed intake [19]. Hosseini-Vashan et al. [20] reported that dietary supplementation with 5% dried tomato pomace increased body weight and decreased feed conversion ratio in broilers under heat stress. Similarly, Wang et al. confirmed that lycopene improved growth performance and antioxidant capacities in Arbor Acres broilers [11]. Wu et al. [2] also found that lycopene improved average daily gain and reduced feed conversion ratios. Consistent with these findings, our findings indicated that Lyc supplementation significantly improved the ADG of broilers from days 29 to 56. Furthermore, the 250 mg/kg Lyc treatment increased the ADFI of birds from days 1 to 28.

Antioxidant enzyme systems play a crucial role in maintaining redox homeostasis in poultry, influencing cell signaling, genetic expression, and stress responses [21]. Key parameters of antioxidant capacity include T-AOC, SOD, GSH-Px, CAT, and MDA [22]. Lyc, as a lipophilic unsaturated carotenoid, could exert antioxidant stability by scavenging reactive oxygen species and inhibiting singlet oxygen [1]. Wang et al. [11] demonstrated that Lyc improved the serum concentrations of GSH-Px, SOD, and T-AOC of Arbor Acres broilers on day 42. Other studies have shown that lycopene increases the hepatic contents of T-AOC and GSH-Px but decreases MDA in birds [23]. Hosseini-Vashan et al. [20] found that the 5% tomato pomace supplementation increased the levels of GSH-Px and decreased MDA in broiler chickens. Arain et al. [24] reported reduced MDA levels in broiler muscle tissue following lycopene treatment. Consistently, our present study revealed that the 250 mg/kg Lyc treatment significantly increased the concentration of hepatic T-AOC and CAT. Moreover, H&E and TEM images revealed reduced liver inflammation in Lyc-treated birds. Lyc250 and Lyc500 significantly decreased the expression of hepatic *Bax*, a key apoptosis-promoting gene. In addition, a study confirmed that high Bax expression and formation could induce the death of cells [25], but more trials should be conducted to investigate the specific signaling pathway for Lyc treatment to decrease Bax expression. These findings indicated that Lyc administration enhances antioxidant capacity and reduces hepatic inflammation and apoptosis in broilers.

Immunoglobulins and pro-inflammatory cytokines, including IgA, IgM, IgY, IL-6, IL-18, and TNF-α, are essential components of the avian immune system [26]. Lycopene supplementation has been shown to boost immune function in yellow-feather broilers [12]. Additionally, dietary Lyc has been reported to enhance immune responses by increasing the indices of immune organs in poultry [27]. Sun et al. [23] found that Lyc improved immune organ development in both hens and their progeny. In our study, Lyc500 significantly increased serum IgY levels, whereas Lyc250 elevated IL-10 and reduced IL-1β levels, reflecting enhanced immune modulation.

The intestinal tract is a crucial organ for nutrient digestion and absorption in poultry. The structural and functional integrity of the intestinal epithelium is essential, with epithelial barriers and gut microbiota playing pivotal roles. Villus height and crypt depth are key morphological indicators of nutrient absorption capacity [28]. Lycopene has been reported to increase small intestinal villus length in hens and their offspring [23]. Wu et al. [2] observed increased villus height in the jejunum and ileum of Arbor Acres broilers following Lyc supplementation. In this study, Lyc treatment positively influenced ileal morphology, with evidence of restored intestinal structure. Lycopene may exert these effects by stimulating digestive enzyme secretion, including alkaline phosphatase, which is linked to epithelial differentiation and improved nutrient absorption [29,30]. Moreover, Lyc decreases intestinal epithelium oxidative injury by reducing ROS and modulating the Keap1/Nrf2 signaling pathway [31].

Intestinal microbiota contributes significantly to digestion, barrier integrity, immune modulation, and disease prevention [32,33]. Wu et al. [2] reported no significant impact of lycopene on cecal microbial diversity (Sobs, Shannon, Simpson, and Chao1 indices), consistent with our findings on α-diversity. However, Gaafar et al. demonstrated that tomato pomace could inhibit pathogenic bacteria, such as *Staphylococcus aureus* [34]. Additionally, oxidized β-carotene was shown to reduce *Clostridium perfringens* and mitigate pathogen-induced intestinal damage [35]. In our previous study, Lyc administration significantly altered cecal microbial composition by increasing *Fischerella* and *Paenactinomyces* in heat-stressed yellow-feather broilers [36]. In the present study, Lyc increased cecal *Bacteroides uniformis*, which is not only capable of utilizing β-glucans but also shares these glycans with intestinal bacteria for potentially maintaining gut microbial homeostasis [37]. Microbiota-derived SCFAs also play critical roles in host physiology, serving as energy sources and modulating the gut–brain axis [38]. Our results demonstrate that Lyc supplementation increases cecal butyric acid concentrations in broilers. The *Ruminococcus* group was reported to produce short-chain fatty acids [39]. The Lyc150 and Lyc250 treatments increased *Ruminococcus sp_g_Ruminococcus* in the present trial, which supported the findings of the above studies. Changes in SCFA levels are closely linked to microbial composition, indicating that lycopene positively influences gut health by modulating both microbiota and their metabolites. A study showed that the LYC treatment improved the intestinal integrity and increased the contents of SCFAs, which are closely related to inner immunity [40].

## 5. Conclusions

Dietary Lyc treated as one antioxidant at a dosage of 250 mg/kg increased the growth performance, enhanced the hepatic antioxidant capacity and intestinal morphology, and modulated the cecal microflora of yellow-feather broilers. Considering the ideal usage dosage and timeframe, more longer-term trials combined with transcriptomics or proteomics should be conducted to analyze the molecular mechanism of Lyc in antioxidant and microbial regulation in the future.

## Figures and Tables

**Table 1:** Composition and nutrient levels of the basal experimental diet (air-dried).

Item	Stage (d)	
	1–28	29–56
composition (%)		
corn	52	60.3
soybean meal	24.7	14
extruded soybean	6	5
corn lees	4	8
corn protein powder	2	4
corn bran		0.6
soybean oil	3.5	2.8
limestone	1.3	1.3
fermented soybean meal	2.5	2.5
vitamin and mineral ^1^	4	4
total	100.00	100.00
nutrition level ^2^		
AME (Kcal/kg)	3050	3125
CP (%)	21	18.2
L-lysine HCl (%)	1.25	0.97
DL-methionine (%)	0.55	0.44
cysteine (%)	0.89	0.76
threonine (%)	0.92	0.74
tryptophan (%)	0.23	0.19
Ca (%)	0.86	0.76
available *p* (%)	0.59	0.48

^1^ Vitamin and mineral premix (s^1^) that supplied each kg of feed with vitamin A, 1500 IU; vitamin D3, 200 IU; vitamin E, 10 IU; vitamin K, 35 g; vitamin B1, 1.5 mg; vitamin B2, 3.5 mg; vitamin B6, 3 mg; vitamin B12, 10 μg; pantothenic acid, 10 mg; nicotinic acid, 30 mg; biotin, 0.15 mg; folic acid, 0.5 mg; choline chloride, 1000 mg; iron, 80 mg; copper, 8 mg; manganese, 60 mg; zinc, 40 mg; selenium, 0.15 mg; and iodine, 0.18 mg. ^2^ Values analyzed from the analysis of the experimental diets.

**Table 2:** Liver cell apoptosis-related genes of yellow-feather broilers.

Gene	Position	Prime Sequence (5′-3′)	Length
*GADPH*	Forward	AGAACATCATCCCAGCGTCC	20
	Reverse	CGGCAGGTCAGGTCAACAAC	20
*Caspase-3*	Forward	GGCTCCTGGTTTATTCAGTCTC	22
	Reverse	ATTCTGCCACTCTGCGATTT	20
*Caspase-9*	Forward	ACAGTGGCAGGGTCCTCAAACAGA	24
	Reverse	GTCACGCAGGGCAAAGAAACTCAG	24
*Bcl-2*	Forward	TCGCGCCGCTACCAGAGGGACTTC	24
	Reverse	CCGGTTGACGCTCTCGACGCACAT	24
*Bax*	Forward	ATCGTCGCCTTCTTCGAGTT	20
	Reverse	ATCCCATCCTCCGTTGTCCT	

**Table 3:** Effects of lycopene on the growth performance of yellow-feather broilers from d 1 to 56.

Item	Con	Lyc150	Lyc250	Lyc500	SEM	*p*-Value
1–28 d						
ADG/g	24.73	25.18	25.22	24.98	0.344	0.493
ADFI/g	42.59 ^b^	44.31 ^ab^	45.32 ^a^	43.78 ^ab^	0.738	0.021
F/G	1.80	1.73	1.77	1.72	0.028	0.053
29–56 d						
ADG/g	41.24 ^b^	47.07 ^a^	47.23 ^a^	47.34 ^a^	1.660	0.007
ADFI/g	111	112.5	119.1	117.3	4.450	0.270
F/G	2.80	2.55	2.71	2.61	0.084	0.057
1–56 d						
ADG/g	33.53	36.1	36.82	36.90	1.845	0.070
ADFI/g	67.14	72.23	71.18	71.42	0.148	0.328
F/G	2.52	2.25	2.38	2.46	1.412	0.108

Note: ADG represents average daily gain, ADFI represents average daily feed intake, and F:G represents the feed/gain ratio. Con represents birds fed a basal diet, Lyc150 represents birds fed a basal diet with 150 mg of lycopene/kg added, Lyc250 represents birds fed a basal diet with 250 mg of lycopene/kg added, and Lyc500 represents birds fed a basal diet with 500 mg of lycopene/kg added. Different lowercase letters represent significantly different means, *p* < 0.05. *n* = 6 per group.

## Data Availability

The original contributions presented in this study are included in the article/Supplementary Materials. Further inquiries can be directed to the corresponding author.

## Supplementary Materials

The following supporting information can be downloaded at: https://www.mdpi.com/article/10.3390/ani15142108/s1, Figure S1: Effects of lycopene on the chao1 (A) and simpson index (B) of caecal microlfora in yellow-feather broilers.

## Abbreviations

The following abbreviations are used in this manuscript:

Lyc	Lycopene
T-AOC	Total antioxidant capacity
CAT	Catalase
V/C	Villus-to-crypt ratio
H&E	Hematoxylin–eosin
SEM	Scanning electron microscopy
ADG	Average daily gain
ADFI	Average daily feed intake
F:G	Feed/gain
SOD	Superoxide dismutase
MDA	Malondialdehyde
GSH-Px	Glutathione peroxidase
TEM	Transmission electron microscopy
SCFAs	Short-chain fatty acids
OTUs	Operational taxonomic units
PCA	Principal component analysis
PCoA	Principal coordinate analysis
LEfSe	Linear discriminant analysis effect size
ANOVA	Analysis of variance
